# Oral Cancer Awareness among Undergraduate Dental Students and Dental Surgeons: A Descriptive Cross-sectional Study

**DOI:** 10.31729/jnma.4847

**Published:** 2020-02-29

**Authors:** Pratibha Poudel, Ritesh Srii, Vinay Marla

**Affiliations:** 1 Department of Oral and Maxillofacial Pathology, Kathmandu University School of Medical Sciences, Dhulikhel, Nepal; 2 Department of Oral Pathology, Penang International Dental College, Penang, Malaysia

**Keywords:** *awareness*, *dental students*, *oral cancer*

## Abstract

**Introduction:**

The incidence of oral cancer is rising due to overindulgence in tobacco chewing and smoking. Its detection in early stage makes it more amenable to treatment and helps to reduce associated morbidity. However, most cases are diagnosed at later stage due to lack of awareness about oral cancer and associated risk factors. This study aims to observe the oral cancer awareness among undergraduate dental students and dental surgeons of three dental institutions of Nepal.

**Methods:**

This descriptive cross-sectional study was conducted in three dental institutions of Nepal from January 2019 to May 2019. Convenience sampling method was used. The data was collected from 508 participants through questionnaire adopted from Carter and Ogden. Point estimate at 95 % Confidence Interval was calculated along with frequency and proportion for binary data. Statistical analysis was done using Statistical Package for the Social Sciences.

**Results:**

Our study showed that 120 (23.6%) of the participants were well informed about the clinical appearance of oral cancer at 95% confidence interval (19.91-27.29%). Most of the participants i.e. 457 (89.96%) and 395 (77.75%) were aware that smoking and chewing tobacco were most commonly recognized risk factors. Only 200 (39.37%) participants were aware that non-healing ulcer is considered as the changes associated with oral cancer. Three hundred and forty-four (67.7%) said they have no knowledge about the prevention and detection of oral cancer.

**Conclusions:**

Our study exhibited the apparent lack of awareness in some aspects of oral cancer and highlights the need of enhancing the undergraduate dental syllabus.

## INTRODUCTION

Oral cancer is considered as one of the major health problems in Asia.^1^ Most of the people are unaware of signs and symptoms of potentially malignant oral lesions (PMOL) and reach hospital after advanced symptoms appear leading to delay in diagnosis and treatment of disease.^2^ The higher morbidity and mortality in oral cancer is due to delay in diagnosis rather than its aggressiveness.^3^

It has been observed that five-year survival rate of oral cancer is only 50%, which can be improved to 80% if the lesion is diagnosed at an early stage.^4^ The existing research has found that high number of general dental practitioners lack sufficient knowledge in recognizing clinical signs of oral cancer which can also be attributed to most cancers diagnosed at later stages.^2^

This study aims to observe the oral cancer awareness among undergraduate dental students and dental surgeons of three dental institutions of Nepal.

## METHODS

This descriptive cross-sectional study was carried out in three different dental institutions of Nepal (Kathmandu University School of Medical Sciences, Kantipur Dental College and Peoples Dental College) representing both Kathmandu University (KU) and Tribhuvan University (TU) from January 2019 to May 2019. The ethical approval was obtained from the Institutional Review Committee of Kathmandu University School of Medical Sciences (IRC-KUSMS No 127/18) and permission was obtained from the respective institution prior to starting the study. Informed consent was obtained from the participants before starting the study-related procedure. Students of BDS third year, fourth year, fifth year, interns and dental surgeons were included in the study whereas students unwilling to participate were excluded from the study. The following formula was used to calculate the sample size considering the prevalence rate as 50%.

n = Z^2^ × p × (1-p) / e^2^

   = (1.96)^2^ × 0.5 × 0.5 / (0.05)^2^

   = 384.16

   = 385

Where,

n= required sample size,
Z= 1.96 for Confidence Interval at 95%,
p= prevalence, 50%,
e= margin of error as 5%,

Taking 20% as a non-response rate, the minimum sample size becomes 462. Thus, a total of 508 participants were included in the study by using a convenience sampling method. Oral cancer awareness among undergraduate dental students and dental surgeons were assessed by means of a questionnaire adopted from the study conducted by Carter and Ogden.^2^ The questionnaire comprised of eleven questions; both open and closed-ended, related to identification and treatment of oral cancer. The participants were given ten minutes to give their responses after which the questionnaires were taken back. The data was then entered in an excel sheet and descriptive statistical analysis was done using SPSS software version 23.0.

## RESULTS

A total of 508 participants were included in the study. Among them, 124 (24.4%) were from the third year,

113 (22.2%) were from the fourth year and 115 (22.6%) were from the fifth year. Interns and dental surgeons were 140 (27.6%) and 16 (3.1%) respectively. Since the sample size for dental surgeons was very less, their responses were combined in the intern group for analysis. Out of 508 participants, 102 (20.1%) were males and 406 (79.9%) were females (Table 1).

**Table 1:** Distribution of subjects in the study.

Variables	n (%)
**Gender**	
Male	102 (20.1)
Female	406 (79.9)
**Year**	
Third year	124 (24.4)
Fourth year	113 (22.2)
Fifth year	115 (22.6)
Intern	140 (27.6)
Dental surgeon	16 (3.1)

A majority of the respondents, i.e. 394 (77.6%) participants responded that they examined the patient’s oral mucosa routinely. As the academic year progressed the frequency of examination was found to be increased, the maximum being among interns and dental surgeons 150 (29.52%) (Table 2).

**Table 2:** Participants response regarding examination of patients oral mucosa routinely.

Groups	Do you examine a patient’s oral mucosa routinely?		
	Yes n (%)	No n (%)	Total n (%)
Third year Fourth year	36 (7.08)	88 (17.32)	124 (24.4)
Third year Fourth year	95 (18.7)	18 (3.54)	113 (22.2)
Fifth year	113 (22.24)	2 (0.39)	115 (22.6)
Intern and Dental surgeon	150 (29.52)	6 (1.18)	156 (30.7)
Total	394 (77.6)	114 (22.4)	508 (100)

Out of 114 students who replied “no” to question one, 81 (66.9%) said that they would screen the oral mucosa if the patients were at high-risk category whereas 33 (8.3%) students said that though the patient was at high-risk category they wouldn’t screen the oral mucosa. Next, an open-ended question was asked regarding the risk factors for oral cancer. A wide range of responses were obtained (Table 3).

**Table 3:** Risk factors for oral cancer identified by the participants.

Risk factors	n (%)
Smoking	457 (89.96)
Chewing tobacco	395 (77.75)
Alcohol	106 (20.86)
Viral infection	72 (14.17)
Genetic factors	62 (12.20)
Dental factors	
Poor oral hygiene	53 (10.43)
Chronic trauma from sharp cusp	
1. UV radiation	44 (8.66)
Dietary factors	
High carbohydrate diet	19 (3.74)
Vitamin B12 defciency	
Immunosuppresion	11 (2.1)
Systemic diseases	2 (0.39)

Most of the participants identified smoking 457 (89.96%) and chewing tobacco 395 (77.75%) as risk factors. An overwhelming, 490 (96.5%) participants said that after graduation they would advise their patients about risk factors for oral cancer. However, 18 (3.5%) students responded with “no” for the same question. Among these 18 students, 14 were from the third year and four were from the fourth year. The next question was “Have you had the opportunity to examine patients with oral lesions?” The response to this question showed that as the clinical year progressed in the dental school, the students got more opportunities to examine patients with oral lesions. It was 7 (5%) among the third years, 12 (10%) among the fourth years, 41 (35%) among the fifth year and 70 (50%) among interns. As regard to the clinical appearance of oral cancer, very few participants thought that they were very well informed. None of the students from the fourth year responded as very well informed. Majority of the students; 62 (50.0%) from third years were poorly informed. Overall only 9 (1.8%) of entire students were very well informed, 120 (23.6%) were well informed, 248 (48.8%) were adequately informed and 131 (25.8%) were poorly informed (Table 4).

**Table 4:** Participants response on clinical appearance of oral cancer.

As regards the clinical appearance of oral cancer, do you feel?					
Groups	well informed n (%)	very well informed n (%)	adequately informed n (%)	poorly informed n (%)	Total n (%)
Third year	27 (21.8)	3 (2.4)	32 (25.8)	62 (50.0)	124 (100.0)
Fourth year	16 (14.2)	0 (0.0)	85 (75.2)	12 (10.6)	113 (100.0)
Fifth year	40 (34.8)	3 (2.6)	44 (38.3)	28 (24.3)	115 (100.0)
Intern and Dental surgeon	37 (23.7)	3 (1.9)	87 (55.8)	29 (18.6)	156 (100.0)
Total	120 (23.6)	9 (1.8)	248 (48.8)	131 (25.8)	508 (100.0)

The question “What changes within the mouth would you associate with oral cancer?” was again an open-ended question and several responses were recorded for this (Table 5). The most common response was found to be “non-healing ulcer, whereas the least common was paraesthesia.

The next question was “When you have graduated where you would refer a patient if you suspected an oral malignancy?” The opinions for this question was fairly divided, with 241 (47.4%) participants responded as oral and maxillofacial surgeons (OMFS) whereas 116 (22.8%) participants felt that they would like to take consultation of both oral medicine and OMFS. Seventy (13.8%) of the respondents said oral medicine as their point of referral.

On the aspect of knowledge regarding the prevention and detection of oral cancer, 113 (91.1%) of third-year students had no knowledge followed by 105 (67.3%) interns and dental surgeons. Only 164 (32.3%) of the entire sample had replied yes for this question whereas 344 (67.7%) said they have no knowledge about this.

Almost all the participants 504 (99.2%) said that they would like more information or teaching on oral cancer with seminar being the most preferred method. Other methods of health education like information pack, lectures, and a combination of methods were all equally preferred. It was also noted that the most health education was required by interns and dental surgeons as compared to the students.

**Table 5:** Participants response regarding changes in mouth associated with oral cancer.

Oral Changes	n (%)
Non-healing ulcer	200 (39.37)
Change in color	137 (26.96)
Red and white lesion	133 (26.18)
Swelling	105 (20.66)
White lesion	32 (6.29)
Red lesion	20 (3.93)
Tooth mobility	18 (3.54)
Hyperkeratosis mucosa	17 (3.34)
Bleeding	10 (1.96)
Lymphadenopathy	6 (1.18)
Induration	6 (1.18)
Pain	5 (0.98)
Chronic infection	2 (0.39)
Necrosis	1 (0.19)
Paraesthesia	1 (0.19)

## DISCUSSION

Early diagnosis of oral cancer is considered to be the key factor in reducing the mortality and morbidity rates associated with it.^5^ In some patients, oral cancer is preceded by PMOL along with histopathological evidence of dysplasia. Diagnosing the lesion in this stage might reduce the frequency of oral malignancy, increase patients survival rate and improve quality of life.^6^

The results of our study showed that out of 508 participants, 79.9% were females while males being only 20.1%. Most of the participants of our study routinely examined the oral mucosa of the patients; and not surprisingly this is higher as the academic year progresses because with this, there is increased exposure of students to the clinical cases as well as improvement in the assessment of cases based on experience. Thirty-three students from our study said that they wouldn't screen the oral mucosa even if the patient were at high-risk category. In a study conducted by Carter and Ogden in the United Kingdom, one out of 109 dental students replied that he/ she would not examine the oral mucosa of high-risk patients. This highlights the facts that undergraduate students from developing country like Nepal could be lagging behind in regard to the knowledge and awareness about oral cancer compared to those of developed country.^7^ Regarding risk factors of oral cancer, most of the participants identified smoking and chewing tobacco and 96.5% mentioned that after graduation they would advise their patients about these risk factors. Halawany et al. conducted a study to evaluate awareness of oral cancer and perception of tobacco use among dental students at all study levels in four Asian countries. More than 95% of students from all four countries identified the association between tobacco smoking and oral cancer.^8^ This could be attributed to the awareness campaigns against tobacco being conducted worldwide on various social and electronic media. However, awareness regarding other risk factors associated with oral cancer were found to be alarmingly low. Based on the results of our study, we found that only 20% or less of the study participants were aware of other risk factors associated with oral cancer. It was found that only 20.86% were aware of alcohol as a risk factor, 14.17% considered viral infection, 12.20% considered genetic factors, 10.43% considered dental factors, 8.66% considered UV radiation and only 3.74% of the respondents considered diet as risk factors for development of oral cancer. Cancer is a multi-factorial disease and a thorough understanding of all the risk factors is important to understand the role of these in the pathogenesis of oral cancer and develop effective prevention strategies. Also, only 3.5% of students said that they would not advise their patients about these risk factors. This indicates that still more emphasis has to be given to encourage the students so that by the time they graduate all the students develop the enthusiasm to advise their patients about this aspect of oral cancer.

The dental syllabus under both TU and KU has been designed in such a way that as the clinical year progresses; there is an increased exposure to clinical postings and community camps. Thus, interns and final year students have more clinical exposure compared to the third year, and this was true in our study as well. Regarding the clinical appearance of oral cancer, none of the students from the fourth year responded as ‘very well informed’; about half of the total participants mentioned as ‘adequately informed’; and one-fourth of them mentioned as ‘poorly informed’. The common mucosal changes identified by the participants were non-healing ulcer, change in colour, mixed red and white lesions and swelling. As mentioned by Bagan et al, the most common clinical presentation of oral cancer in initial stage was red and white lesion; while for advanced cases, the participants responded as ulcers and lumps.^9^ It was found in our study that the awareness of changes in oral mucosa associated with oral cancer was quite low. Only 39.37% identified non-healing ulcer as manifestation of oral cancer, which is a cause of worry since dentists are among the first professionals who have an opportunity to screen the oral mucosa. Awareness of other manifestations of oral cancer was even low with change in color (26.96%), red & white lesions (26.18%), swelling (20.66%) and awareness of other manifestations less than 10% respectively. Oral cancer is a dynamic pathology with no specific presentation and rate of progression. Many instances, these can present as innocuous lesion and may be missed during early screening leading to diagnosis at later stages.^10^ A thorough understanding of the oral changes associated with oral cancer is significant in the early diagnosis of these lesions. The above-discussed findings are suggestive that the undergraduate dental students and dental surgeons have inadequate knowledge regarding cancer. It shows the need for further education on this topic. More focus could be given in problem-based learning (PBL) which enables students to increase their knowledge, identify the problems and develop effective application skills on solving these problems.^11^ Most of the participants responded ‘OMFS’ and ‘Oral Medicine’ as their point of the referral. This is in accordance with a similar study done by Carter et al. and Ogden et al. among undergraduate dental students in the United Kingdom and Iran respectively.^2,12^ Nevertheless, these two studies also suggested that this could be a bias due to the word “oral” being a part of both these specialist terminologies. This clearly shows a lack of clear understanding of oral cancer as a whole, since the ability to refer a patient at the right time to the right specialist can turn out to play a key role in early diagnosis of oral cancer. Reports from developed countries have highlighted the medico-legal implications associated with failure to make proper referrals.^13^

Despite their increased exposure to patients in clinics, 67.3% of interns and dental surgeons said that they have no knowledge regarding the prevention and detection of oral cancer. More than 90% of third-year students agreed to this statement. Our results are in agreement with the study from Sri Lanka which showed that 70% of dentists wanted more training in terms of oral cancer screening.^14^ This indicates the knowledge gap that dental surgeons are having especially in these parts of the world where oral cancer incidence is said to be the highest.^15^ Almost all the participants mentioned that they would like more information or teaching on oral cancer with a seminar being the most preferred method. The conventional method of teaching by didactic lectures seems to be less preferred by today’s generation. Presenting a seminar by students they require a lot of effort from their side. They need to search in-depth about the topic before presenting which will certainly increase their knowledge and understanding about the topic. Apart from this, the audio-visual aids used in the seminar further make it easy to understand the topic in a better way. It is appreciable that students are willing to upgrade their knowledge of oral cancer by presenting in the seminars. This can be facilitated by incorporating a seminar on oral cancer as a part of the teaching-learning process in various subjects like oral pathology, oral medicine, and oral surgery. This way, the students will get to know and refine their knowledge about oral cancer every year of dental school.

We also noted in our study that most of the interns and dental surgeons seek for health education compared to the students. This could be due to the fact that interns and dental surgeons do not have the added burden for studying for exams and they have developed the understanding to identify the areas of defciencies, which could affect their clinical practice in the future. This can be addressed by involving the interns and dental surgeons for routine case presentations and discussions at the hospital, encouraging them to attend continuing professional development (CPD) programs regularly and also by assigning them to present seminars. Mentoring the young and budding dental professionals is the right way to tackle the menace of tobacco and early identification of PMOL and oral cancers.

To summarize, it can be concluded that the overall awareness and knowledge of oral cancer is inadequate among the undergraduate dental students and dental surgeons in Nepal. The findings of this study highlight the strengthening of our existing undergraduate dental curriculum for tackling this deadly disease effectively. The topic of oral cancer should be given more credit hours and should include regular field visits to local cancer centres for providing more exposure to the students on this topic. An integrated teaching module involving the department of oral medicine and radiology, oral pathology, and oral surgery for an effective understanding of the various aspects of this disease. Nevertheless, our study doesn’t represent data from all the dental colleges of Nepal. Also having only two open-ended questions to evaluate student's knowledge regarding oral cancer could be another limitation.

## CONCLUSIONS

The overall awareness of oral cancer is inadequate among the undergraduate dental students and dental surgeons in our study. However, this can be achieved through continuing dental education program for dental surgeons and fortifying the dental syllabus of BDS by incorporating structured teaching program with emphasis on identifying risk factors, early signs and symptoms of oral premalignant and malignant lesions.
